# Summary of the best evidence on blood flow restriction training for patients after anterior cruciate ligament reconstruction surgery

**DOI:** 10.3389/fresc.2026.1806660

**Published:** 2026-08-04

**Authors:** Yao Wang, Fei Wang, Bin Xu, Xiaobing Yin

**Affiliations:** 1 School of Medicine, Tongji University, Shanghai, China; 2 Department of Nursing, Shanghai Tenth People’s Hospital, Shanghai, China

**Keywords:** anterior cruciate ligament reconstruction, blood flow restriction training, evidence summary, evidence-based nursing, rehabilitation

## Abstract

**Objective:**

This study systematically retrieved, evaluated, and synthesized the best available evidence on blood flow restriction training for patients following anterior cruciate ligament reconstruction, aiming to provide evidence-based guidance for clinical implementation of this training modality among healthcare professionals.

**Methods:**

A systematic search was conducted to identify the best available evidence on blood flow restriction training for patients undergoing anterior cruciate ligament reconstruction. The search encompassed multiple types of literature, including clinical guidelines, expert consensus statements, systematic reviews, meta-analyses, and evidence summaries. The following databases were searched up to April 2025: UpToDate, BMJ Best Practice, and the Cochrane Library. Two researchers independently screened the retrieved literature, assessed methodological quality using standardized tools [Appraisal of Guidelines for Research and Evaluation II (AGREE II) for guidelines and Joanna Briggs Institute (JBI) critical appraisal checklists for other study types], and graded the level of evidence according to prespecified criteria.

**Results:**

A total of 18 studies were included in this review, consisting of two clinical guidelines, one evidence summary, two expert consensus statements, and 13 systematic reviews. In total, 21 evidence points were extracted and categorized into 11 thematic categories: multidisciplinary team involvement, training principles, applicable populations, contraindications, assessment indicators, training equipment, training protocols, training intensity, training frequency and duration, real-time assessment and monitoring, and safety precautions.

**Conclusions:**

The evidence summary on blood flow restriction training for patients following anterior cruciate ligament reconstruction is both scientifically rigorous and comprehensive, providing a robust evidence-based foundation for clinical implementation. Healthcare providers should tailor the training to individual patient circumstances to ensure both safety and therapeutic efficacy.

**Systematic Review Registration:**

identifier ES20257963.

## 1 Introduction

Anterior cruciate ligament reconstruction (ACLR) is the standard treatment for anterior cruciate ligament (ACL) injury and serves as a critical intervention for maintaining tibiofemoral sagittal alignment and rotational stability (1). With the increasing popularity of national fitness initiatives, the annual incidence of ACL injury has risen steadily. Epidemiological data indicate that approximately 200,000 individuals sustain ACL injuries each year (2). Surgical intervention enables anatomical reduction and effectively restores knee joint stability in affected patients (3). However, suboptimal postoperative rehabilitation and insufficient functional exercise mean that only 65% of patients are able to return to their preinjury functional level (4). Moreover, postoperative limb swelling and pain frequently lead to exercise-related fear, reducing patients’ tolerance for moderate- to high-intensity rehabilitation and thereby compromising the overall effectiveness of rehabilitation programs. Consequently, identifying an exercise modality that delivers superior rehabilitation outcomes at low intensity is of considerable clinical importance. Blood flow restriction (BFR) training, also known as KAATSU training, is a rehabilitation and conditioning technique that partially restricts arterial inflow and fully occludes venous return by applying external pressure to the proximal aspect of the limb using inflatable cuffs or elastic bandages. This method has been shown to enhance muscle strength, increase muscle mass, and improve muscular endurance, and has been widely adopted in medical rehabilitation, fitness training, and competitive sports (5, 6). At present, considerable heterogeneity exists in BFR training protocols for patients following ACLR, with no consensus yet established regarding optimal training intensity, pressure parameters, treatment duration, or other critical variables (7). To address this gap, the present study synthesizes the available evidence on BFR training for post-ACLR patients, with the dual aim of providing practical guidance for nurses in implementing this intervention and offering a reference for enhancing the quality of postoperative rehabilitation. This project has been registered with the Evidence-Based Nursing Center at Fudan University (Registration No: ES20257963).

## 2 Methods

### 2.1 Research purpose

This study was structured according to the PIPOST framework to formulate an evidence-based inquiry (8). The inclusion criteria were defined as follows: Population (P): patients following anterior cruciate ligament reconstruction; Intervention (I): blood flow restriction training (BFRT); Professionals (P): healthcare professionals in clinical or community settings; Outcomes (O): knee range of motion (ROM), muscle strength, and cross-sectional area of knee-surrounding muscles (including the quadriceps femoris and hamstrings); Setting (S): outpatient departments, inpatient wards, or community hospitals; and Type of evidence (T): guidelines, expert consensus statements, systematic reviews, meta-analyses, expert opinions, and evidence summaries. This study was reported in accordance with the PRISMA-ScR guidelines to ensure methodological rigor and transparency.

### 2.2 Search strategy

The literature search was conducted sequentially from top to bottom according to the evidence resource “6S” hierarchy model. We first searched for preappraised, synthesized evidence using systems such as UpToDate and BMJ Best Practice. Next, we searched databases including the Joanna Briggs Institute Evidence-Based Practice (JBI EBP) Database and the Cochrane Library for review abstracts and full reviews (i.e., systematic reviews, meta-analyses, and evidence summaries). Using “anterior cruciate ligament” and “blood flow restriction therapy” as keywords, we also searched the websites of guideline development organizations and professional associations, including the Guidelines International Network (GIN), the National Guideline Clearinghouse (NGC), the National Institute for Health and Clinical Excellence (NICE), the Scottish Intercollegiate Guidelines Network (SIGN), the Registered Nurses’ Association of Ontario (RNAO), and the Clinical Practice Guidelines Website of the Canadian Medical Association. The search employed a combination of subject headings and free-text terms. The main search terms were “anterior cruciate ligament,” “anterior cruciate ligament reconstruction,” and “anterior cruciate ligament injuries” for the condition, combined with “blood flow restriction therapy,” “BFR,” “blood flow restriction exercise,” “blood flow restriction,” “vascular occlusion,” and “occlusion training” for the intervention. The following databases were searched: Cochrane Library, CINAHL, PubMed, Web of Science, Embase, PEDro, the Chinese Medical Association Website, China National Knowledge Infrastructure (CNKI), Wanfang Database, and VIP Database. The detailed search strategies for each database are provided in Supplementary Table S1.

### 2.3 Literature inclusion and exclusion criteria

The inclusion criteria were defined as follows: (1) population: patients aged 18 years or older who had undergone anterior cruciate ligament reconstruction; (2) availability of full text; (3) latest version of guidelines; (4) language: Chinese or English; and (5) publication types: guidelines, expert consensus statements, clinical standards, systematic reviews, meta-analyses, evidence summaries, and clinical practice guidelines.

The exclusion criteria were defined as follows: (1) literature that had been updated or republished; (2) literature consisting of interpretations or translations of guidelines; (3) research proposals, drafts, reports, and conference abstracts; (4) literature rated as low quality; and (5) gray literature, including but not limited to dissertations, theses, preprints, clinical trial registry records, government reports, white papers, and unpublished raw data. Gray literature was excluded because such sources typically lack peer review, exhibit variable methodological quality, and may not provide sufficient detail for evidence synthesis. Moreover, preliminary searches indicated that no relevant gray literature met the preestablished quality threshold for inclusion in this evidence summary.

### 2.4 Quality evaluation of the literature

The Appraisal of Guidelines for Research and Evaluation II (AGREE II) instrument was used to evaluate the methodological quality of included guidelines (9). This tool comprises six domains encompassing 23 items, each scored on a seven-point Likert scale ranging from 1 (“strongly disagree”) to 7 (“strongly agree”). Based on the standardized domain scores, guidelines were classified into three recommendation grades: Grade A (recommended) required a standardized score of ≥60% across all six domains; Grade B (recommended with modifications) required scores of ≥30% in at least three domains, with scores below 60% in some domains; and Grade C (not recommended) required scores below 30% in at least three domains.

The JBI Critical Appraisal Checklist for Text and Opinion Papers was used to assess this category of evidence. The checklist consists of six evaluation criteria, each rated as “Yes,” “No,” “Unclear,” or “Not applicable.” Final decisions regarding inclusion, exclusion, or the need for further clarification were determined through panel discussion.

For systematic reviews and meta-analyses, we used the 2016 version of the JBI Critical Appraisal Checklist for Systematic Reviews and Research Syntheses. This tool comprises 11 items, each rated as “Yes,” “No,” “Unclear,” or “Not applicable.” Studies with nine or more “Yes” responses were classified as high quality; those with six to eight “Yes” responses were classified as moderate quality; and those with fewer than six “Yes” responses were classified as low quality.

Clinical decision and evidence summaries were evaluated using the Critical Appraisal for Summaries of Evidence (CASE) tool, which consists of 10 items with response options “Yes,” “Partly yes,” and “No.” All quality assessments were conducted independently by two researchers trained in evidence-based methodology. In the event of disagreement, a third evidence-based expert was consulted to facilitate discussion and reach final consensus.

### 2.5 Evidence extraction, integration, and evaluation

Two researchers independently extracted, categorized, and synthesized evidence from the final selected literature. The 2014 version of the evidence grading criteria developed by the Australian JBI Centre for Evidence-Based Medicine was used to classify the evidence into five levels, with higher levels indicating more rigorous study designs (Level 1a representing the highest and Level 5c the lowest). In addition, the research team applied the JBI FAME (Feasibility, Appropriateness, Meaningfulness, Effectiveness) framework to evaluate each piece of evidence and assign a recommendation grade of either Grade A (strong recommendation) or Grade B (weak recommendation) (10). During evidence synthesis, high-quality reviews with low risk of bias were prioritized as core evidence, whereas studies with moderate or severe methodological limitations were treated as tentative evidence with reduced weighting. Ultimately, our final recommendations were based not solely on the consistency of empirical findings but on a comprehensive integration of result stability and the methodological quality of all eligible evidence.

## 3 Results

### 3.1 General characteristics of the included literature

**Figure 1:**
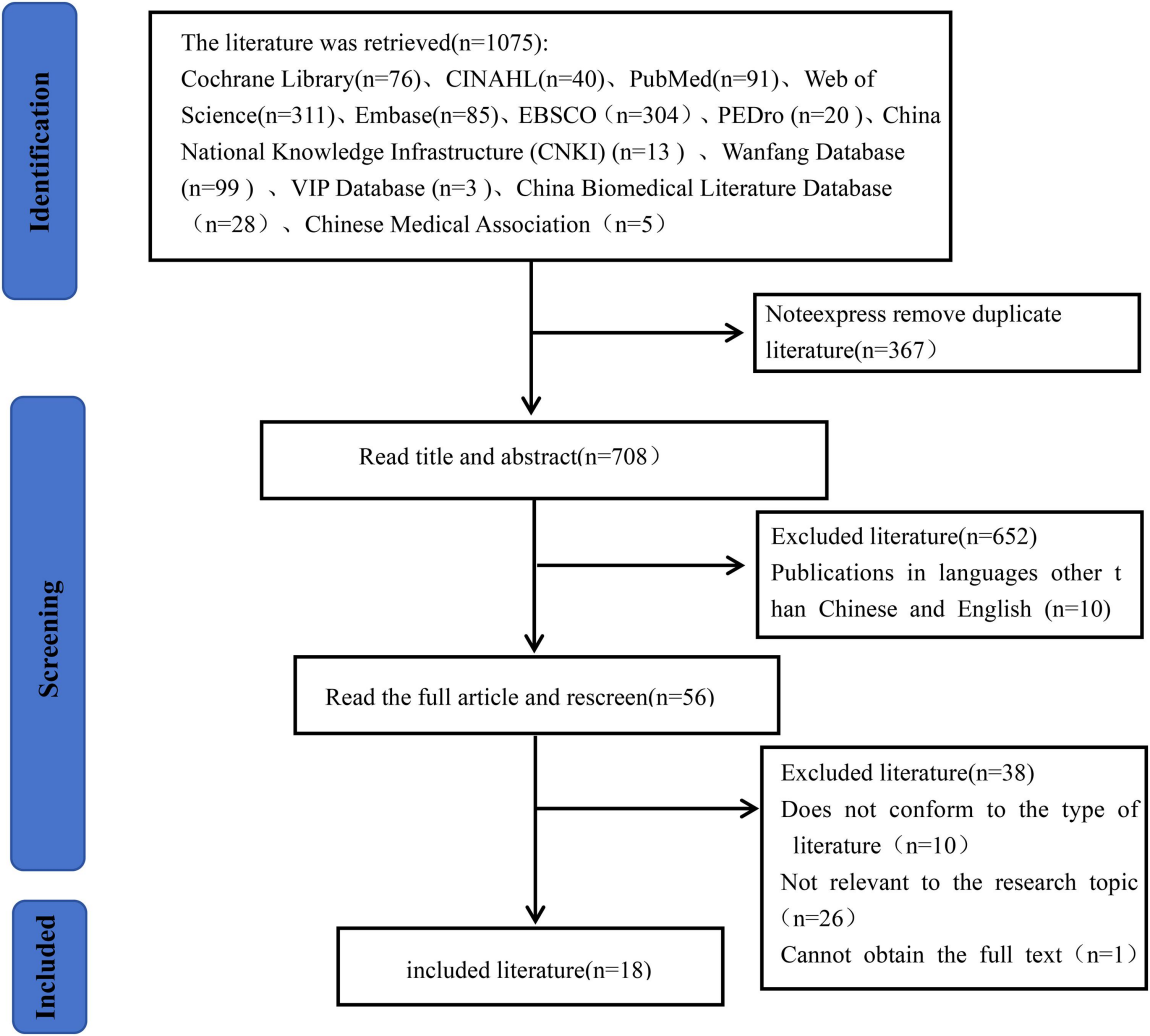
Flowchart of literature screening.

A total of 1,075 records were identified through the initial search. After importing the records into NoteExpress and removing duplicates, 708 articles remained. Two team members independently screened the titles and abstracts of each article. Following the exclusion of articles without full-text access and those deemed irrelevant, 56 articles remained for full-text quality assessment. After quality appraisal, 18 articles were finally included, comprising two guidelines (4, 11), 13 systematic reviews (8, 12–23), one evidence summary (24), and two expert consensus statements (7, 25). The literature screening process is illustrated in Figure 1, and the general characteristics of the included studies are summarized in Table 1.

**Table 1:** General characteristics of the included literatures (*n* = 18).

Inclusion in the literature	Year of publication (year)	Source of the article	Type of literature	Literature theme
Cognetti et al. (25)	2022	PubMed	Expert consensus	The application of blood flow restriction training in rehabilitation and return to sports
Kotsifaki et al. (4)	2023	PubMed	Guidelines	Clinical practice guidelines for rehabilitation after anterior cruciate ligament reconstruction
Lorenz et al. (11)	2021	Web of Science	Guidelines	Blood flow restriction training
Liu et al. (24)	2025	CNKI	Summary of Evidence	Rehabilitation intervention after arthroscopic anterior cruciate ligament reconstruction surgery
DePhillipo et al. (7)	2018	PubMed	Expert consensus	The role of blood flow restriction training after knee joint surgery
Rodríguez et al. (12)	2024	PubMed	Systematic review	The acute and chronic effects of blood flow restriction training on patients after anterior cruciate ligament reconstruction
Glattke et al. (13)	2021	PubMed	Systematic review	Recovery and rehabilitation of patients after anterior cruciate ligament reconstruction surgery
Spada et al. (14)	2022	PubMed	Systematic review	Blood flow restriction training preserves knee flexion and extension torque after anterior cruciate ligament reconstruction
Colombo et al. (15)	2024	PubMed	Systematic review	Comparison of blood flow restriction training intervention and standard rehabilitation after anterior cruciate ligament injury
Koc et al. (16)	2022	PubMed	Systematic review	The impact of low-load blood flow restriction training on patients after anterior cruciate ligament reconstruction
Fernández et al. (17)	2024	PubMed	Systematic review	Effects of blood flow restriction training on patients before and after anterior cruciate ligament reconstruction surgery
Lin et al. (18)	2024	PubMed	Meta-analysis	The effect of low-load blood flow restriction training on muscle volume in patients after anterior cruciate ligament reconstruction surgery
Chang et al. (19)	2023	PubMed	Meta-analysis	Effects of different cuff pressures on muscle strength
Lu et al. (8)	2020	PubMed	Systematic review	Effects of perioperative blood flow restriction training on patients after anterior cruciate ligament reconstruction
Wengle et al. (20)	2022	PubMed	Meta-analysis	The impact of blood flow restriction training on patients undergoing knee joint surgery
Li et al. (21)	2025	PubMed	Meta-analysis	The effect of blood flow restriction training on muscle strength and knee joint function in patients after anterior cruciate ligament reconstruction
Gopinatth et al. (22)	2025	PubMed	Meta-analysis	Blood flow restriction training promotes the recovery of patients after anterior cruciate ligament reconstruction surgery
Ogrezeanu et al. (23)	2025	PubMed	Meta-analysis	Effects of blood flow restriction training on patients undergoing anterior cruciate ligament reconstruction: preoperative and postoperative outcomes

### 3.2 Quantitative assessment of overlap in original research

To quantify the degree of primary study overlap among the included systematic reviews, we constructed a citation overlap matrix based on 13 systematic reviews and 59 unique primary studies, and calculated the corrected covered area (CCA). The analysis revealed a total citation frequency of 102 and a CCA of 6.07%. This value falls within the moderate overlap range, indicating that a certain degree of original study duplication exists across the included systematic reviews, which may pose a risk of duplicate weighting in the evidence synthesis.

### 3.3 Quality evaluation results of the included literature

In the quality appraisal process, the interrater intraclass correlation coefficient values for all assessments exceeded 0.750, indicating a high level of consistency and good reliability among the evaluators.

#### 3.3.1 Quality evaluation results of the clinical guidelines

This study included two guidelines (4, 11), both of which scored ≥60% in five AGREE II domains and were rated as Grade B. Accordingly, these guidelines were considered to be of moderate methodological quality and were included in this review. The results of the guideline quality assessment are presented in Table 2.

**Table 2:** Guidelines’ quality evaluation results.

Guideline	Standardized scores in various domains (%)								
	Scope and purpose	Stakeholder involvement	Rigor of development	Clarity of presentation	Applicability	Editorial independence	≥60%	≥30%	Quality evaluation
Kotsifaki et al. (4)	100	77.78	96.43	100	58.33	100	5	6	B
Lorenz et al. (11)	100	83.33	74.07	86.67	58.33	91.67	5	6	B

#### 3.3.2 Quality evaluation results of the expert consensus

Quality assessment was performed on two expert consensus documents [7.19]. Item 6 of DePhillipo (7) was evaluated as “unclear,” while the remaining items were all rated “yes.” Therefore, this expert consensus was considered high quality and included in the summary. The evaluation results are presented in Table 3.

**Table 3:** Guidelines’ quality evaluation results.

Expert consensus	①	②	③	④	⑤	⑥
Cognetti et al. (25)	Yes	Yes	Yes	Yes	Yes	Yes
Lorenz et al. (11)	Yes	Yes	Yes	Yes	Yes	Unclear

① Is the source of the opinion clearly identified? ② Does the source of the viewpoint have standing in the field of expertise? ③ Are the interests of relevant groups the central focus of opinions? ④ Is the stated position the result of the analysis process? Is the viewpoint expressed logically? ⑤ Do you have any references to the existing literature? ⑥ Is there any inconsistency between the viewpoints presented and the previous literature?

#### 3.3.3 Quality assessment results of evidence summary

One evidence summary was included in this study (24). The quality appraisal showed that one item was rated as “unclear,” whereas the remaining items were all rated “Yes.” Overall, the methodological quality was considered satisfactory, and the evidence summary was therefore included in the final analysis. Detailed evaluation results are presented in Table 4.

**Table 4:** The quality evaluation of evidence summary.

Items	Liu Jingwen et al. (24)
1. Is the summary specific in scope and application?	Yes
2. Is the authorship of the summary transparent?	Yes
3. Are the reviewer(s)/editor(s) of the summary transparent?	Yes
4. Are the search methods transparent and comprehensive?	Yes
5. Is the evidence graded and is the system transparent and translatable?	Yes
6. Are the recommendations clear?	Yes
7. Are the recommendations appropriately cited?	Yes
8. Are the recommendations current?	Yes
9. Is the summary free of possible bias?	Unclear
10. Can this summary be applied to your patient(s)?	Yes

#### 3.3.4 Quality evaluation results of the system evaluation

This study included 13 systematic reviews published over the past decade (8, 12–23), all of which addressed blood flow restriction training. As shown in Supplementary Table S2, all 13 reviews were rated as high quality.

### 3.4 Summary and description of evidence

Through evidence extraction and integration, 21 pieces of best evidence were synthesized across ten thematic domains: training principles, applicable populations, contraindications, assessment content, training equipment, training content, training intensity, training frequency, outcome evaluation, and safety precautions (Table 5).

**Table 5:** Summary of the best evidence on blood flow restriction training for patients after anterior cruciate ligament reconstruction surgery.

Evidence topic	Evidence content	Evidence level	Recommendation grade
Multidisciplinary team	1. Form a multidisciplinary rehabilitation team in which orthopedic surgeons and nurses work collaboratively with physical therapists to develop treatment plans for patients (7).	Level 5b	B
Training principles	2. During training, the following principles should be followed: continuous monitoring, gradual adjustments, individualized programs, screening for contraindications, and symptom monitoring (8, 23). It is recommended that training be supervised by a professional to ensure proper use of the cuff and correct execution of the exercises (20).	Level 2b	A
Applicable population	3. Suitable for patients in the early rehabilitation phase following anterior cruciate ligament (ACL) reconstruction, knee arthroscopy, or fracture fixation (4, 11, 12, 17), and particularly suitable for individuals who, due to significant postoperative pain, are unable to tolerate high-intensity training, or those who experience muscle atrophy, insufficient muscle strength, or functional limitations (12, 15, 20, 23). It may also be used for preoperative rehabilitation prior to ACL reconstruction (20).	Level 1b	A
Contraindications	4. Absolute contraindications: patients with severe cardiovascular disease (hypertension, a history of deep vein thrombosis, or those at high risk of thrombosis; peripheral arterial disease), skin irritation or infection, open wounds, pregnancy, renal insufficiency, or lymphatic system disorders (4, 12, 14, 20).	Level 1b	A
	5. Relative contraindications: patients with severe localized swelling of the limbs, joint effusion, or those at high risk of skeletal muscle injury, such as those with severe osteoporosis, a recent history of fractures, coagulation disorders, diabetes with peripheral neuropathy, or those who are sensitive to pain or unable to tolerate cuff compression (12).	Level 2b	B
Evaluation content	6. Pretraining assessment: Screen the patient's medical history to identify any contraindications for training; determine whether the postoperative tissue-healing phase is suitable for training; and assess the patient's knee range of motion, quadriceps and hamstring muscle strength, muscle atrophy, and subjective function. In addition, evaluate the patient's 1RM and arterial occlusion pressure (AOP) to provide a basis for determining the compression pressure (4, 11).	Level 1b	A
	7. Post-training assessment: The assessment primarily consists of objective measures and subjective experiences. Objective measures include muscle volume, muscle strength, range of motion in the knee joint, joint stability, limb symmetry, and wound healing. Subjective experiences include patient-reported outcomes and psychological assessments (11, 20).	Level 1b	B
	8. Safety Assessment: The assessment includes vascular response tests, such as distal pulse checks, oxygen saturation monitoring, and skin examinations, to determine whether any adverse reactions have occurred, such as transient numbness, skin indentations, delayed muscle soreness, deep vein thrombosis, or rhabdomyolysis. If numbness, tingling, or severe pain occurs, the compression band should be loosened immediately (4).	Level 1b	A
Training equipment	9. Pressure devices include manually or electrically pumped inflatable pressure bands and elastic pressure bands; pneumatic adjustment systems are typically preferred. Supporting equipment includes pressure monitoring devices and low-resistance training tools (4).	Level 1b	A
	10. The recommended cuff width is 5–6 cm for the upper extremities and 9–14 cm for the lower extremities. The cuff should be placed on the proximal end of the moving limb. For patients who have undergone anterior cruciate ligament reconstruction, the cuff should be placed 10 cm above and below the knee joint (18). A protective pad should be used inside the cuff to prevent skin damage. For the lower extremities, the cuff should be placed on the proximal thigh, such as at the distal third of the groin (8, 11).	Level 1b	A
Training content	11. Training Modalities: Training can be combined with low-load resistance training, aerobic exercise, passive exercise, and neuromuscular electrical stimulation; currently, the most common approach is to combine it with resistance training (25).	Level 5b	A
Training intensity	12. Methods for determining cuff pressure: The five most common methods for determining cuff pressure include selecting a fixed pressure, using a percentage of systolic blood pressure, using limb circumference, using a tension intensity scale, or using a percentage of arterial occlusion pressure (11).	Level 1b	A
	13. Pressure levels: It is recommended to use the AOP to determine the pressure required for complete occlusion. Doppler ultrasound or a pulse oximeter may be used to monitor distal arteries and assess pressure levels. Measurements should be taken at the same site to avoid factors such as body position from affecting the arterial occlusion pressure (11). Since training outcomes are influenced by cuff pressure characteristics, intermittent compression is recommended (19).	Level 1b	A
	14. Low-load blood flow restriction training is recommended to be performed at 20%–40% of 1RM, with the compression pressure set at 40%–80% of the limb occlusion pressure, as an alternative to traditional strength rehabilitation (18).	Level 1b	A
	15. For pressure settings, it is recommended to use individualized pressure (rather than fixed values) and progressive loading, gradually increasing the load based on the patient's postoperative tolerance. The pressure range is typically set at 40–180 mmHg for the upper limbs, and 50–200 mmHg for the lower limbs. It is recommended that a training load of 20%–40% of 1RM be used as the initial treatment regimen for early rehabilitation in patients following anterior cruciate ligament reconstruction (4, 11, 22). Each session should be limited to no more than 10–20 min, and the reperfusion time is typically 30–180 s (4, 20).	Level 1b	A
Training frequency and duration	16. Patients can begin training in the early postoperative period (1–2 weeks), with a recommended starting frequency of 2–3 times per week. During the intermediate rehabilitation phase (4–12 weeks), this can be combined with traditional resistance training, with a recommended frequency of 2–3 times per week. In the late rehabilitation phase (≥12 weeks), it can be used as a supplementary exercise, with a recommended frequency of 1–2 times per week (4, 21, 23).	Level 1b	A
	17. It is recommended to perform 3–5 sets per workout, with the first set consisting of 30 repetitions and subsequent sets of 15 repetitions each, or an average of 15–30 repetitions per set, with a rest interval of 30–60 s between sets (11, 14, 23). The recommended set sequence consists of four sets: the first set with 30 repetitions, and the second, third, and fourth sets with 15 repetitions each. A rest period of 30–60 s is recommended between sets, and training is recommended 2–3 times per week (7).	Level 1b	A
Evaluation and monitoring	18. Primary outcome measures include knee flexion and extension torque, functional recovery, muscle strength, muscle cross-sectional area, and knee function, which can be assessed through muscle strength testing or muscle circumference measurements. Secondary outcome measures include patient pain scores [e.g., visual analogue scale (VAS)], ROM, and swelling severity (11, 17).	Level 1b	B
	19. Blood flow restriction training combined with low-load training is as effective as high-load training (70% 1RM) in increasing muscle cross-sectional area and muscle strength; it results in less postexercise soreness than high-load training and can significantly improve pain levels, IKDC scores, and isokinetic muscle strength, with particularly pronounced effects at 80% LOP (13).	Level 2b	A
	20. Blood flow restriction training does not appear to significantly improve quadriceps strength or muscle mass in the early postoperative period (≤3 weeks); however, it has been shown to significantly improve knee function during mid-term follow-up and thus plays a positive role (21).	Level 1b	B
Safety precautions	21. During training, patients with ACLR may experience reactions such as local ischemia at the surgical site, lactic acid accumulation, and muscle damage; symptoms are typically mild and reversible (19). Common adverse reactions include pain, delayed-onset muscle soreness, and increased heart rate or blood pressure. Serious risks include nerve paralysis, ischemic injury, and thrombosis (18). Termination criteria include numbness, dizziness, abnormal pain, or pallor (11).	Level 1b	A

## 4 Discussion

### 4.1 Establish a multidisciplinary collaboration model to jointly guide patients in standardized training

Evidence items 1 and 2 indicate that BFR training for patients following ACLR should be delivered under the supervision of orthopedic surgeons and rehabilitation therapists. The establishment of a multidisciplinary team is essential to ensure standardized, evidence-based training delivery and to optimize the quality of rehabilitation nursing care. These recommendations are supported by multiple randomized controlled trials drawn from systematic reviews and expert consensus statements, with an evidence level of 2. In international practice, BFR training for ACLR patients is predominantly led by rehabilitation therapists and clinicians, whereas multidisciplinary collaboration remains in its early stages. Standardized protocols for the specific implementation of BFR training in this population are still lacking. Therefore, it is recommended that training protocols be developed collaboratively by a multidisciplinary team.

Evidence item 2 further specifies the training principles to be observed during the rehabilitation process. Orthopedic surgeons are expected to assess patients’ preoperative conditions—including graft type and joint stability—to determine the appropriate timing for initiating BFR training and to identify any contraindications. Rehabilitation therapists are responsible for designing individualized training regimens, determining pressure parameters, selecting appropriate movements and frequencies, and instructing patients on the correct use of equipment. During training, nurses should monitor limb circulation and promptly report any abnormal vital signs.

### 4.2 Understanding the rehabilitation phase and identifying the key points of pre-training assessment are prerequisites for safe training

Evidence items 7–9 emphasize the necessity of pre- and post-training assessments, as well as that of ongoing safety evaluations, for patients undergoing blood flow restriction training. These recommendations, derived from systematic reviews, clinical guidelines, and expert consensus statements, delineate the core components of training-related assessment, which serve three essential functions: forming the basis for individualized rehabilitation protocols, ensuring correct and effective training execution, and functioning as a prerequisite for safe implementation. Given the presence of contraindications, systematic screening of eligible populations prior to training is a critical first step. Clinicians must evaluate the tissue-healing status of ACLR patients to determine their suitability for early intervention. Previous studies have suggested that BFR training may be applicable to frail populations—including older adults and athletes—undergoing clinical rehabilitation (8). For ACLR patients who cannot tolerate high-load training in the early postoperative phase, this modality has been shown to enhance muscle strength, improve functional outcomes, and alleviate pain (4). When administered according to standardized protocols, BFR training is generally safe; however, contraindications must be carefully observed. Evidence items 5 and 6 specify that training should be avoided by patients with severe cardiovascular disease, high deep venous thrombosis (DVT) risk or active thrombosis, diabetes, or coagulation disorders. Accordingly, for ACLR patients undergoing BFR training for the first time, nursing staff should collaborate with clinicians and rehabilitation therapists to conduct comprehensive pretraining screening and reinforce safety monitoring, thereby ensuring safe and effective implementation.

### 4.3 Formulating appropriate exercise parameters serves as the safety guarantee for the rehabilitation of ACLR patients

Pressure parameters and exercise parameters are the two most important components of BFRT and are closely related to rehabilitation outcomes and patient safety following ACLR. Evidence items 9–17 summarize the pressure and exercise parameters of BFRT, covering aspects such as pressure devices, application sites, pressure levels, training frequency, exercise intensity, and duration. Much of this evidence is derived from high-quality literature and provides practical guidance for clinical implementation.

With regard to the timing of training initiation, a divergence exists between guideline recommendations and expert opinions. Guideline 1 recommends that patients begin training in the early postoperative period (1–2 weeks), particularly those who are unable to tolerate high-load training due to pain or joint swelling. In contrast, expert opinion (4) suggests a more conservative approach, recommending initiation at 2–4 weeks postoperatively. Based on the principles of evidence grading, guidelines carry a higher level of evidence; therefore, this study adopts the recommendations of Guideline 1. Studies have shown that BFRT can serve as an effective alternative to high-intensity training for ACLR patients, and that its implementation in the early postoperative period is both safe and beneficial to the rehabilitation process.

The training equipment primarily consists of compression devices and an occlusion cuffs. Narrower cuffs require higher pressures to achieve arterial occlusion, whereas wider cuffs can achieve the same effect at lower pressures. Therefore, current clinical recommendations suggest that cuff width should be selected individually based on the patient's training purpose, target site, and limb circumference. The cuff should be placed at the proximal end of the limb being exercised, such as the proximal thigh or the distal third of the groin. A protective sleeve may be placed on the training limb prior to use to reduce the risk of skin friction injuries.

With regard to pressure assessment, multiple studies recommend using limb occlusion pressure (LOP), which is determined based on individual patient characteristics—including vascular elasticity, limb circumference, and blood pressure—to avoid limb ischemia caused by excessive pressure and to prevent reduced training efficacy resulting from insufficient pressure. In clinical practice, Doppler ultrasound or a pulse oximeter is recommended for monitoring distal arterial pressure values; measurements should be taken at the same anatomical site each time to avoid interference from factors such as body position.

The American College of Sports Medicine (ACSM) states that high-load resistance training (≥70% 1RM) is an effective method for promoting muscle strength gains; however, this training modality is not suitable for patients who have undergone ACLR. Due to postoperative limb pain, swelling, and knee joint impairment, ACLR patients often find it difficult to complete high-load training. Excessive loading in the early postoperative period not only damages the graft and increases the risk of reinjury but also causes severe pain and subjective discomfort, thereby increasing the likelihood of tissue damage (26). Therefore, low-load training should be prioritized in the early postoperative period. Combining BFRT with low-load resistance training has been shown to significantly enhance training outcomes (12). A low-load intensity of 20%–40% 1RM is recommended, with compression pressure set at 40%–80% LOP. Intermittent compression is preferred, with reperfusion intervals of 30–180 s. The training process should follow the principle of gradual progression: In the early postoperative period (1–2 weeks), training should be performed 2–3 times per week; during the midrecovery phase (4–12 weeks), BFRT should be combined with traditional resistance training at a frequency of 2–3 times per week; in the late recovery phase (>12 weeks), BFRT may be used as a supplementary training method at a frequency of 1–2 times per week. Each training session should consist of 3–5 sets, with the first set comprising 30 repetitions and subsequent sets 15 repetitions each, with 30–60 s of rest between sets (7). Low-load training alleviates joint stress caused by high-load exercises in the early postoperative period, thereby improving patients’ exercise tolerance. With regard to the duration of intervention, studies suggest that initiating BFRT 1–3 weeks before surgery and continuing it for 8–14 weeks postoperatively yield more significant outcomes.

Currently, there are no unified standards for BFRT exercise parameters for ACLR patients. Systematic reviews and expert opinions are conflicting, and there are no specific guidelines for this population. Therefore, training programs must be individually tailored based on the patient's postoperative stage, degree of swelling, pain level, graft type, and other individual characteristics. Further randomized controlled trials are needed to establish a more evidence-based, stratified system for selecting BFRT protocols.

### 4.4 Critical appraisal of evidence strength and certainty

Although the evidence included in this review is primarily classified as Level 1 or Level 2 according to the JBI evidence preclassification system, several methodological limitations must be carefully considered when interpreting the strength of the recommendations from this study. The first issue is that of sample size. Most of the original randomized controlled trials (RCTs) included in the systematic reviews had relatively small sample sizes (typically fewer than 40 participants per group), which may lead to overestimation of effect sizes or failure to detect clinically meaningful differences (Type II errors). For example, Colombo et al. (15) noted that among the 12 RCTs included in their meta-analysis, only 3 had sufficient sample sizes to detect clinically meaningful differences in quadriceps strength. This limitation is particularly relevant for secondary outcome measures (such as patient-reported functional status), as smaller effect sizes may be underestimated in these measures. Second, it is difficult to conduct double-blind studies. In BFRT studies, blinding participants and intervention providers is particularly challenging due to the nature of the intervention: Participants can feel the pressure from the cuff, and therapists are aware of the pressure level being applied. Although outcome assessors can be blinded, operational and detection biases remain a concern. This limitation is not unique to our current evidence base but represents a systemic challenge prevalent throughout the BFRT literature, which should be acknowledged when interpreting study results. Third, publication bias remains. Of the 13 systematic reviews included, only 4 (30.8%) formally assessed publication bias using methods such as the Egger’s test or funnel plots. Among the studies that conducted such assessments, two reported asymmetry suggestive of potential publication bias, indicating that studies with positive results may be overrepresented in the literature. This is of particular concern given that the field is dominated by smaller-scale studies, which are therefore more susceptible to publication bias. The fourth issue is that of inconsistency in outcome measures. The included systematic reviews employed a wide variety of outcome measures, and this heterogeneity limits the comparability of results across different studies.

In intervention studies, the methods used to determine cuff pressure vary considerably across studies. These methods include fixed pressures (e.g., 150 or 200 mmHg) and percentages of LOP or arterial occlusion pressure (AOP) (typically 40%–80%). Although individualized pressure based on LOP/AOP has become the preferred method, inconsistencies remain regarding measurement techniques (Doppler ultrasound vs. pulse oximetry vs. auscultation) and timing of measurement (preoperative vs. early postoperative vs. before each training session). Chang et al. (18) reported that, compared with fixed pressure, personalized pressure settings may yield a more significant increase in muscle strength [standardized mean difference (SMD) =0.42，95% CI 0.18–0.66]; however, this conclusion is based on indirect comparisons and lacks direct confirmation. There are also differences in training frequency and duration. Training frequency ranged from 2 to 6 times per week (mean: 3.2 times/week), and intervention duration ranged from 4 to 12 weeks (mean: 7.6 weeks). Lu et al. (8) noted that at least 12 training sessions are required to achieve measurable gains in muscle strength, but this conclusion was derived from a *post hoc* analysis of a single RCT. Since the follow-up periods in relevant studies rarely exceeded 6 months, it remains unclear whether these long-term improvements are sustainable.

### 4.5 Comparison of evidence summary with major guidelines

The best evidence synthesized in this study is broadly concordant with the practice recommendations of mainstream guidelines in terms of the overarching principles of early prevention, multidimensional assessment, and integrated intervention strategies. Nevertheless, our comprehensive analysis further expands upon existing guidelines by providing relatively detailed protocols for BFRT—including specific parameters for pressure settings, training frequency, and patient selection. For other interventions, such as high-load resistance training combined with early postoperative rehabilitation, the current evidence base remains insufficient to support definitive recommendations. Therefore, we maintain a cautious stance consistent with major guidelines and identify these gaps in evidence as priority areas for future research.

### 4.6 Limitations

This evidence summary has inherent limitations. Several methodological limitations of this evidence synthesis merit acknowledgment. First, the predominant reliance on secondary sources carries the inherent risk of overlapping primary study inclusion. We sought to minimize this by systematically comparing reference lists across the included reviews and calculating the CCA(6.07%) to quantify the overlap, which indicated a moderate degree of primary study overlap. Further *post hoc* analysis based on the citation matrix revealed that the sources of overlap were diffusely distributed, and no single review was found to have exclusive contributions to the highly cited primary studies, partially mitigating the potential impact of duplicate weighting on our conclusions. Nevertheless, readers should interpret our findings with this methodological limitation in mind. Second, the restriction of our search to Chinese- and English-language publications, together with the deliberate exclusion of gray literature, may have introduced both language and publication biases. Third, considerable heterogeneity was observed across the included systematic reviews with respect to BFR protocols and outcome measures, which constrains the comparability of findings and tempers the strength of our recommendations. Fourth, although the included evidence was predominantly classified as JBI Level 1 or 2, methodological shortcomings in the underlying RCTs—including limited sample sizes, inherent difficulties in blinding participants and personnel, and infrequent formal evaluation of publication bias—indicate that the overall certainty of our recommendations is more appropriately characterized as moderate rather than high. Fifth, our search was current only to April 2025, and thus the synthesis does not capture evidence published thereafter. Regular updates will be required to ensure ongoing clinical relevance. Notwithstanding these limitations, we believe this synthesis constitutes the most comprehensive and clinically actionable evidence summary currently available.

## 5 Conclusion

Based on the principles of evidence-based medicine, this study summarizes the best available evidence for BFR training in patients undergoing ACLR, encompassing 21 pieces of evidence across nine dimensions: applicable populations, contraindications, training assessment, training equipment, training content, training intensity, training frequency, efficacy evaluation, and safety precautions. This work provides an evidence-based foundation for healthcare professionals to implement BFR training in ACLR rehabilitation.

However, the specific protocols and strategies for postoperative BFR training in ACLR patients still require further refinement through additional studies of high methodological quality. This study did not include high-quality RCT literature, as the main included sources were systematic reviews and expert opinions, which may have resulted in the omission of some high-quality original studies. Future research should further enrich the evidence base and provide more comprehensive guidance for clinical practice.

In the clinical implementation of BFR training, individualized intervention protocols should be formulated based on the clinical characteristics and individual differences of ACLR patients. Such tailoring will provide more specific guidance for the translation of research evidence into clinical practice.

## Data Availability

The original contributions presented in the study are included in the article/Supplementary Material, further inquiries can be directed to the corresponding author.
