# Supplementary material for: Summary of the best evidence on blood flow restriction training for patients after anterior cruciate ligament reconstruction surgery
Source: Front Rehabil Sci. 2026 Aug 4;7:1806660. doi: 10.3389/fresc.2026.1806660 (PMC13481354; doi:10.3389/fresc.2026.1806660)
Supplement: Supplementary file 1 [file Table1.docx]

**Supplementary Table 1. The literature search strategy**

| **Database** | **Search Strategy** |
| --- | --- |
| **Web of Science** | #1 TS=(ACL OR ACLR OR "anterior cruciate ligament" OR "anterior cruciate ligament reconstruction" OR "anterior cruciate ligament injuries")  #2 TS=("blood flow restriction" OR BFR OR "vascular occlusion" OR "occlusion training" OR "blood flow restriction training" OR kaatsu OR "tourniquet training")  #3 TS=(postoperative rehabilitation OR "muscle strength" OR "muscle atrophy" OR knee torque OR "postoperative recovery" OR knee "functional outcome")  #4 #1 AND #2 AND #3 |
| **Pubmed** | **#1 "Anterior Cruciate Ligament"[MeSH]**  **#2 "Anterior Cruciate Ligament Reconstruction"[MeSH]**  **#3 "Anterior Cruciate Ligament Injuries"[MeSH]**  **#4 (ACL[tiab] OR ACLR[tiab] OR "anterior cruciate ligament"[tiab] OR "anterior cruciate ligament reconstruction"[tiab] OR "anterior cruciate ligament injuries"[tiab])**  **#5 #1 OR #2 OR #3 OR #4**  **#6 "Blood Flow Restriction Therapy"[MeSH]**  **#7 ("blood flow restriction"[tiab] OR BFR[tiab] OR "vascular occlusion"[tiab] OR "occlusion training"[tiab] OR kaatsu[tiab] OR "tourniquet training"[tiab])**  **#8 #6 OR #7**  **#9 (rehabilitation[tiab] OR "muscle strength"[tiab] OR "muscle atrophy"[tiab] OR torque[tiab] OR "functional outcome"[tiab])**  **#10 ("systematic review"[tiab] OR "meta-analysis"[tiab] OR "meta analysis"[tiab] OR "randomized controlled trial"[tiab] OR "RCT"[tiab])**  **#11 #5 AND #8 AND #9 AND #10** |
| **Embase** | **#1'acl':ab,ti OR 'aclr':ab,ti OR 'anterior cruciate ligament':ab,ti OR 'anterior cruciate ligament reconstruction':ab,ti OR 'anterior cruciate ligament injuries':ab,ti OR 'acl reconstruct*':ab,ti OR 'anterior cruciat* ligament reconstruct* surger*':ab,ti OR 'anterior cruciat* ligament repair*':ab,ti OR 'reconstruct* surger*':ab,ti OR 'anterior cruciat* ligament reconstruct*':ab,ti OR 'repair* surger*':ab,ti**  **AND**  **#2'blood flow restriction therapy':ab,ti OR 'bfr':ab,ti OR 'blood flow restriction exercise':ab,ti OR 'blood flow restriction':ab,ti OR 'vascular occlusion':ab,ti OR 'occlusion training':ab,ti OR 'blood flow restriction training':ab,ti OR 'bfr therapy':ab,ti OR 'bfr therapies':ab,ti OR 'therapy, bfr':ab,ti OR 'blood flow* restrict* train*':ab,ti OR 'rehabilitat* train* blood flow* limitat*':ab,ti OR 'blood flow* occlus* train*':ab,ti OR 'inflatabl* compress* train*':ab,ti OR 'muscl* congest* train*':ab,ti OR 'dist* ischemic precondit* train*':ab,ti OR 'hypoxic blood* flow* train*':ab,ti OR 'vascular restrict* train*':ab,ti OR 'pressure train*':ab,ti OR 'train*':ab,ti**  **AND**  **#3'perioperative period':ab,ti OR 'postoperat*':ab,ti OR 'perioperative management*':ab,ti**  **#4 #1AND#2AND#3** |
| **Cochrane Library** | **#1 (Anterior Cruciate Ligament OR Anterior Cruciate Ligament Reconstruction OR Anterior Cruciate Ligament Injuries OR ACL OR ACLR):ti,ab,kw**  **#2 (blood flow restriction therapy OR BFR OR blood flow restriction exercise OR blood flow restriction OR vascular occlusion OR occlusion training):ti,ab,kw**  **#3 (guideline OR meta-analysis OR expert opinion OR consensus OR systematic review OR evidence summary):ti,ab,kw**  **#4 #1 AND #2 AND #3** |
| **CINAHL** | **#1 SU (Anterior Cruciate Ligament OR Anterior Cruciate Ligament Reconstruction OR Anterior Cruciate Ligament Injuries OR ACL OR ACLR)**  **#2 SU (blood flow restriction therapy OR BFR OR blood flow restriction exercise OR blood flow restriction OR vascular occlusion OR occlusion training)**  **#3 SU (guideline OR meta-analysis OR expert opinion OR consensus OR systematic review OR evidence summary)**  **#4 #1 AND #2 AND #3** |
| 中国知网  (China National Knowledge Infrastructure, CNKI) | #1 SU=(' 前交叉韧带 ' + ' 前交叉韧带损伤 ' + ' 前交叉韧带重建术 ' + ' 前交叉韧带重构术 ' + 'ACL 重建术 ' + ' 前十字韧带 ' + ' 前十字韧带重建术 ' + ' 前十字韧带重构术 ')  #2 SU=(' 血流限制训练 ' + ' 血流阻滞训练 ' + ' 加压训练 ')  #3 #1 AND #2 |
| 万方数据  (Wanfang Data) | #1 主题：(前交叉韧带 OR 前交叉韧带损伤 OR 前交叉韧带重建术 OR 前交叉韧带重构术)  #2 主题：(血流限制训练 OR 血流阻滞训练 OR 加压训练)  #3 #1 AND #2 |
| 维普数据 (Weipu Data) | #1 M=(前交叉韧带 OR 前交叉韧带损伤 OR 前交叉韧带重建术)  #2 M=(血流限制训练 OR 血流阻滞训练 OR 加压训练)  #3 #1 AND #2 |
| 中国生物医学文献数据库 (China Biomedical Literature Database) | #1 “前交叉韧带”[常用字段：智能] OR “前交叉韧带损伤”[常用字段：智能] OR “前交叉韧带重建术”[常用字段：智能] OR “前交叉韧带重构术”[常用字段：智能]  #2 “血流限制训练”[常用字段：智能] OR “血流阻滞训练”[常用字段：智能] OR “加压训练”[常用字段：智能]  #3 #1 AND #2 |
