# Supplementary material for: Summary of the best evidence on blood flow restriction training for patients after anterior cruciate ligament reconstruction surgery
Source: Front Rehabil Sci. 2026 Aug 4;7:1806660. doi: 10.3389/fresc.2026.1806660 (PMC13481354; doi:10.3389/fresc.2026.1806660)
Supplement: Supplementary file 2 [file Table2.docx]

**Supplementary Table 2 The quality evaluation of systematic reviews**

| **Items** | **Chang et al(21)** | **Colombo et al.(14)** | **Fraca-Fernández et al.（16）** | **García-Rodríguez et al.(11)** | **Glattke et al（12）** | **Gopinatth**  **et al(24)** | **Koc**  **et al(15)** | **Li**  **et al(23)** | **Lin et al(20)** | **Lu et al(17)** | **Ogrezeanu et al(25)** | **Spada et al（13）** | **Wengle et al（22）** |
| --- | --- | --- | --- | --- | --- | --- | --- | --- | --- | --- | --- | --- | --- |
| *1.Is the review question clearly and explicitly stated?* | Yes | Yes | Yes | Yes | Yes | Yes | Yes | Yes | Yes | Yes | Yes | Yes | Yes |
| *2.Were the inclusion criteria appropriate for the review question?* | Yes | Yes | Yes | Yes | Yes | Yes | Yes | Yes | Yes | Yes | Yes | Yes | Yes |
| *3.Was the search strategy appropriate?* | Yes | Yes | Yes | Yes | Yes | Yes | Yes | Yes | Yes | Yes | Yes | Yes | Yes |
| *4.Were the sources and resources used to search for studies adequate?* | Yes | Yes | Yes | Yes | Yes | Yes | Yes | Yes | Yes | Yes | Yes | Yes | Yes |
| *5.Were the criteria for appraising studies appropriate?* | Yes | Yes | Yes | Yes | Yes | Yes | Unclear | Yes | Yes | Yes | Yes | Yes | Yes |
| *6.Was critical appraisal conducted by two or more reviewers independently?* | Yes | Yes | Yes | Yes | Yes | Yes | Yes | Yes | Yes | Yes | Yes | Yes | Yes |
| *7.Were there methods to minimize errors in data extraction?* | Yes | Yes | Yes | Yes | Yes | Yes | Yes | Yes | Yes | Yes | Yes | Yes | Yes |
| **Items** | **Chang et al(21)** | **Colombo et al.(14)** | **Fraca-Fernández et al.（16）** | **García-Rodríguez et al.(11)** | **Glattke et al（12）** | **Gopinatth**  **et al(24)** | **Koc**  **et al(15)** | **Li**  **et al(23)** | **Lin et al(20)** | **Lu et al(17)** | **Ogrezeanu et al(25)** | **Spada et al（13）** | **Wengle et al（22）** |
| *8.Were the methods used to combine studies appropriate?* | Yes | Unclear | Yes | Unclear | Yes | Yes | Unclear | Yes | Yes | Yes | Yes | Yes | Yes |
| *9.Was the likelihood of publication bias assessed?* | Yes | Unclear | Yes | Yes | Yes | Yes | Yes | Yes | Yes | Yes | Yes | Yes | Unclear |
| *10.Were recommendations for policy and/or practice supported by the reported data?* | Yes | Yes | Yes | Yes | Yes | Yes | Yes | Yes | Yes | Yes | Yes | Yes | Yes |
| *11.Were the specific directives for new research appropriate?* | Yes | Yes | Yes | Yes | Yes | Yes | Yes | Yes | Yes | Yes | Yes | Yes | Yes |
| Quality Rating | high quality | high quality | high quality | high quality | high quality | high quality | high quality | high quality | high quality | high quality | high quality | high quality | high quality |
